# Kinetics of the porcine reproductive and respiratory syndrome virus (PRRSV) humoral immune response in swine serum and oral fluids collected from individual boars

**DOI:** 10.1186/1746-6148-9-61

**Published:** 2013-03-28

**Authors:** Apisit Kittawornrat, Mark Engle, Yaowalak Panyasing, Chris Olsen, Kent Schwartz, Anna Rice, Sergio Lizano, Chong Wang, Jeffrey Zimmerman

**Affiliations:** 1 Department of Veterinary Diagnostic and Production Animal Medicine, College of Veterinary Medicine, Iowa State University, Ames, IA, 50011, USA; 2 Department of Statistics, College of Liberal Arts and Sciences, Iowa State University, Ames, IA, 50011, USA; 3 PIC North America, 100 Bluegrass Commons Blvd, Hendersonville, TN, 37075, USA; 4 IDEXX Laboratories, Inc, Westbrook, ME, 04092, USA

**Keywords:** PRRSV, Oral fluid, ELISA, IgM, IgA, IgG, Antibody kinetics

## Abstract

**Background:**

The object of this study was to describe and contrast the kinetics of the humoral response in serum and oral fluid specimens during acute porcine reproductive and respiratory syndrome virus (PRRSV) infection. The study involved three trials of 24 boars each. Boars were intramuscularly inoculated with a commercial modified live virus (MLV) vaccine (Trial 1), a Type 1 PRRSV field isolated (Trial 2), or a Type 2 PRRSV field isolate (Trial 3). Oral fluid samples were collected from individual boars on day post inoculation (DPI) -7 and 0 to 21. Serum samples were collected from all boars on DPI −7, 0, 7, 14, 21 and from 4 randomly selected boars on DPI 3, 5, 10, and 17. Thereafter, serum and oral fluid were assayed for PRRSV antibody using antibody isotype-specific ELISAs (IgM, IgA, IgG) adapted to serum or oral fluid.

**Results:**

Statistically significant differences in viral replication and antibody responses were observed among the three trials in both serum and oral fluid specimens. PRRSV serum IgM, IgA, and IgG were first detected in samples collected on DPI 7, 10, and 10, respectively. Oral fluid IgM, IgA, and IgG were detected in samples collected between DPI 3 to 10, 7 to 10, and 8 to 14, respectively.

**Conclusions:**

This study enhanced our knowledge of the PRRSV humoral immune response and provided a broader foundation for the development and application of oral fluid antibody-based diagnostics.

## Background

The presence of systemic and locally-produced antibodies in oral fluid has led to its use as a diagnostic specimen for a variety of infectious diseases. In humans, oral fluids have been used in the diagnosis of human immunodeficiency virus (HIV), Hepatitis A, B, and C viruses, measles, mumps and other infectious diseases [1]. In swine, antibodies against a variety of economically significant pathogens have been reported in oral fluids, including classical swine fever virus [2,3], porcine circovirus type 2 [4], porcine reproductive and respiratory syndrome virus [5,6], swine influenza virus [7], transmissible gastroenteritis virus [8], *Actinobacillus pleuropneumoniae*[9], and *E. coli*[10]. In large measure, the kinetics of the antibody response against individual agents has not been described. Therefore, the purpose of present study was to describe and contrast the ontogeny of PRRSV IgM, IgA, and IgG in oral fluids and serum specimens collected from individually housed boars during acute PRRSV infection.

## Results

### PRRSV antibody isotypes in serum

**Figure 1:**
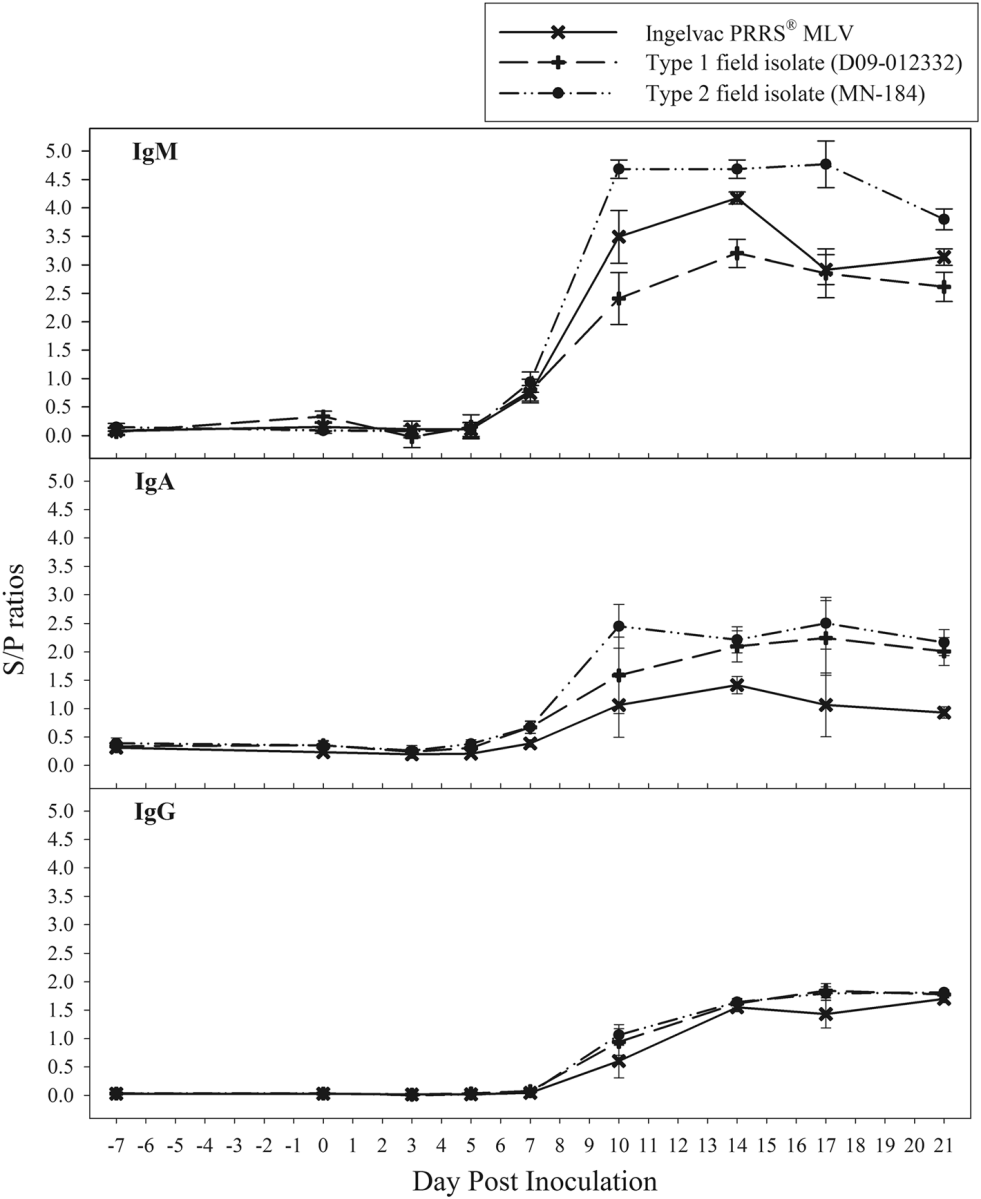
**Kinetics of PRRSV antibody isotypes (IgM, IgA, and IgG) in serum based on responses in 72 boars inoculated with 3 different PRRSV isolates.** Results are reported as mean sample-to-positive (S/P) ratios and standard errors.

The PRRSV antibody isotype (IgM, IgA, and IgG) responses in serum samples are shown in Figure 1 and Table 1. Estimates for DPI −7, 0, 7, 14, 21 were based on data from 72 boars, whereas estimates for DPIs 3, 5, 10, and 17 were based on a subset of 12 animals randomly selected from the 72 boars. Based on pairwise comparisons, statistically significant levels of IgM were detected by DPI 7, peaked at DPI 14, and remained stable through DPI 21. In contrast, statistically significant levels of IgA and IgG were detected by DPI 10, after which they remained stable (IgA) or increased significantly (IgG) through DPI 21. Further analysis showed that IgM and IgA responses were associated with DPI (IgM: *p* < 0.0001, IgA: *p* < 0.0001), trial (IgM: *p* < 0.0001, IgA: *p* < 0.0149), and the interaction between trial and DPI (IgM: *p* < 0.0001, IgA: *p* < 0.0001). In contrast, DPI (*p* < 0.0001) was the only factor associated with the IgG response. Neither the age of the boar at the time of inoculation nor the quantity of oral fluid collected from each boar had a significant effect on IgM, IgA, or IgG.

**Table 1:** Porcine reproductive and respiratory syndrome virus (PRRSV) antibody isotypes (IgM, IgA, and IgG) in oral fluid and serum samples collected from boars over day post inoculation (DPI)

**DPI**	**Serum (mean S/P ratios)**				**Oral fluid (mean S/P ratios)**			
	**Samples**	**IgM**	**IgA**	**IgG**	**Samples**	**IgM**	**IgA**	**IgG**
−7	72	0.10 ^d^	0.35 ^b^	0.03 ^d^	70	0.02 ^g^	0.15 ^g^	0.01 ^i^
0	72	0.19 ^d^	0.31 ^b^	0.03 ^d^	68	0.02 ^g^	0.08 ^g^	0.01 ^i^
1	-	-	-	-	69	0.04 ^g^	0.12 ^g^	0.01 ^i^
2	-	-	-	-	68	0.04 ^g^	0.13 ^g^	0.02 ^i^
3	12	0.05 ^d^	0.23 ^b^	0.01 ^d^	70	0.07 ^g^	0.16 ^g^	0.02 ^i^
4	-	-	-	-	68	0.13 ^g^	0.24 ^g^	0.01 ^i^
5	12	0.12 ^d^	0.30 ^b^	0.02 ^d^	67	0.05 ^g^	0.11 ^g^	0.01 ^i^
6	-	-	-	-	66	0.09 ^g^	0.12 ^g^	0.01 ^i^
7	72	0.82 ^c^	0.58 ^b^	0.05 ^d^	69	0.30 ^f^	0.23 ^g^	0.03 ^i^
8	-	-	-	-	66	1.28 ^e^	0.41 ^f^	0.17 ^h^
9	-	-	-	-	66	2.32 ^c^	0.95 ^e^	0.59 ^g^
10	12	3.56 ^b^	1.70 ^a^	0.87 ^c^	70	2.72 ^b^	1.45 ^b, c^	0.99 ^f^
11	-	-	-	-	70	2.95 ^a^	1.75 ^a^	1.41 ^e^
12	-	-	-	-	70	2.70 ^b^	1.59 ^a, b^	1.78 ^d^
13	-	-	-	-	69	2.64 ^b^	1.64 ^a^	2.05 ^c^
14	72	4.02 ^a^	1.91 ^a^	1.60 ^b^	68	2.32 ^c^	1.56 ^a, b^	2.28 ^b^
17	12	3.51 ^b^	1.94 ^a^	1.69 ^a, b^	69	1.53 ^d^	1.25 ^c, d^	2.63 ^a^
21	72	3.18 ^b^	1.70 ^a^	1.76 ^a^	68	1.02 ^e^	1.14 ^d, e^	2.60 ^a^

^a-i^ Superscripts within columns indicate statistically significant differences among means (Tukey’s Honestly Significant Differences test, *p* < 0.05).

### PRRSV antibody isotype in oral fluid samples

**Figure 2:**
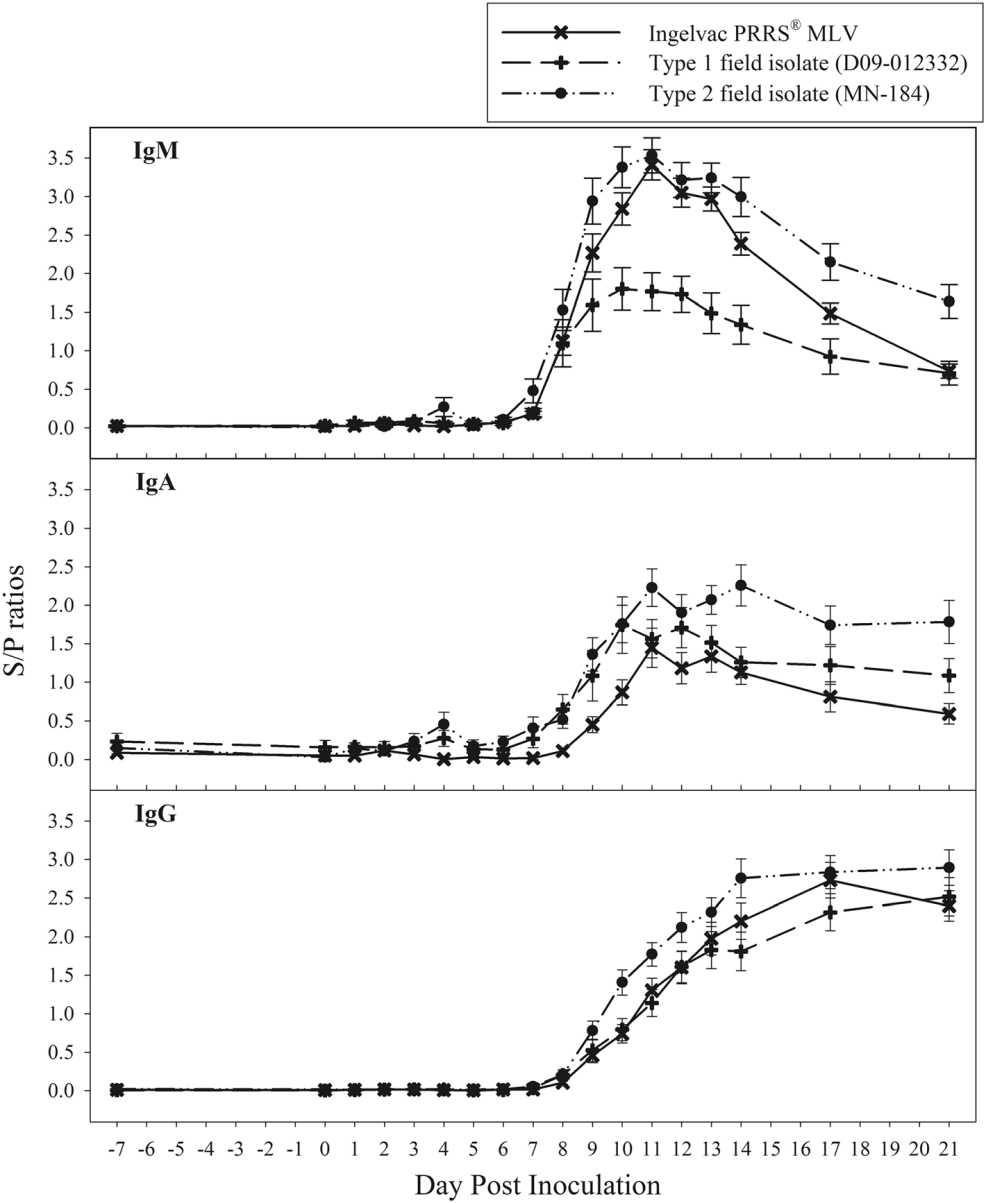
**Kinetics of PRRSV antibody isotypes (IgM, IgA, and IgG) in oral fluid based on responses in 70 boars inoculated with 3 different PRRSV isolates.** Results are reported as mean sample-to-positive (S/P) ratios and standard errors.

The PRRSV antibody isotype (IgM, IgA, and IgG) responses in oral fluid samples are shown in Figure 2 and Table 1 for oral fluid samples collected on DPI −7, 0–14, 17, and 21. Estimates were based on ≥66 oral fluid samples at each sampling point. IgM S/P ratios were statistically significant on DPI 7, peaked at DPI 11, and declined thereafter. Levels of IgA and IgG were significant on DPI 8 and increased thereafter through the end of the experiment (DPI 21). Factors significantly associated with IgM and IgA S/P ratios included trial (IgM: *p* < 0.0001, IgA: *p* = 0.0273), DPI (IgM: *p* < 0.0001, IgA: *p* < 0.0001), oral fluid volume (IgM: *p* = 0.0002, IgA: *p* < 0.0001), and the interaction between trial and DPI (IgM: *p* < 0.0001, IgA: *p* < 0.0001). Factors associated with IgG included DPI (*p* < 0.0001) and the interaction of trial by DPI (*p* < 0.0001). Regardless of isotype, boar age at the time of inoculation had no significant effect on antibody response.

### Comparison of PRRSV antibody responses in serum and oral fluid

**Figure 3:**
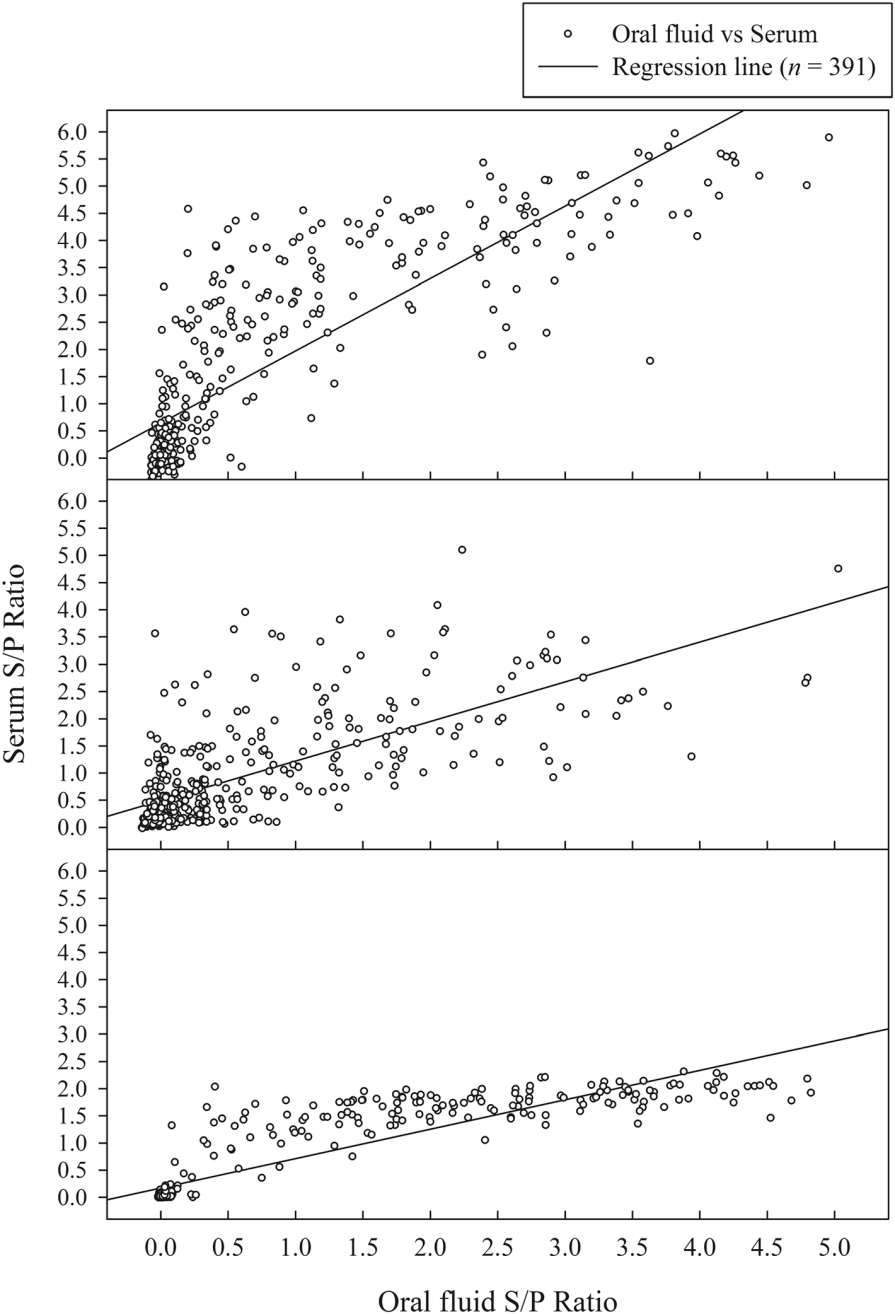
Correlation between serum and oral fluid PRRSV antibody isotypes (IgM, IgA, and IgG) based on results from individual boars.

A comparison of the qualitative antibody response in serum vs. oral fluid found no significant difference in the proportion of ELISA positive results on DPI 0 – 14, 17, and 21 (Table 2). No significant difference was detected in the number of positive results for each pair-wise sample combination (serum vs. oral fluid) by trial, DPI, or trial by DPI. On DPI 21, 100% of serum and oral fluid samples were positive with mean S/P ratios of 1.69 (95% Confidence Interval [CI]: 1.58, 1.79) and 2.60 (95% CI: 2.34, 2.86), respectively. An analysis of the quantitative antibody isotype responses in serum and oral fluid samples using test results from samples collected on DPI −7, 0 – 14, 17, 21 estimated Pearson’s correlation coefficient as r = 0.84, 0.78, and 0.90 for IgM, IgA, and IgG responses, respectively (Figure 3).

**Table 2:** Porcine reproductive and respiratory syndrome virus (PRRSV) serum^a^ and oral fluid^b^ ELISA qualitative results^c^ by day post inoculation (DPI)

**Trial (virus isolate)**	**Sample**	**DPI 0 positive / tested**	**DPI 7 positive / tested**	**DPI 14 positive / tested**	**DPI 21 positive / tested**
Trial 1: (Ingelvac® PRRS MLV)	Oral fluid	0 / 24	0 / 24	24 / 24	24 / 24
	Serum	0 / 24	0 / 24	24 / 24	24 / 24
Trial 2: (Type 1, D09-012332)	Oral fluid	0 / 22	0 / 22	17 / 21	22 / 22
	Serum	0 / 24	0 / 24	22 / 24	24 / 24
Trial 3: (Type 2, MN-184)	Oral fluid	0 / 24	0 / 23	24 / 24	22 / 22
	Serum	0 / 24	0 / 24	24 / 24	24 / 24
Total	Oral fluid	0 / 70	0 / 69	65 / 69	68 / 68
	Serum	0 / 72	0 / 72	70 / 72	72 / 72

^a^ PRRS X3 Ab Test (IDEXX Laboratories, Inc, Westbrook, Maine, USA) performed according to the manufacturer’s instruction.

^b^ PRRS X3 Ab Test (IDEXX Laboratories, Inc, Westbrook, Maine, USA) modified to detect anti-PRRSV antibody in oral fluid specimens [5].

^c^ Samples with S/P ratio ≥ 0.4 were classified as positive.

### Comparison between viremia levels and antibody responses

All serum samples from DPI −7 and 0 (*n* = 144) were PRRSV qRT-PCR negative, whereas all serum samples from boars tested on DPI 3 (*n* = 12) and DPI 7 (*n* = 72) were positive. PRRSV was detected in oral fluids from 7 of 69 boars at DPI 1, 52 of 68 boars at DPI 2, 66 of 70 at DPI 3, and all boars were PRRSV qRT-PCR positive at DPI 4. A comparison of matched samples from individual boars showed that oral fluid was equal to serum for the detection of PRRSV at DPI 7 and more likely to be positive than serum on DPI 14 and 21. These data are reported in detail elsewhere [11].

To evaluate the association between viremia and antibody responses, cumulative serum and oral fluid PRRSV qRT-PCR (log_e_ geq/μl) and antibody isotype (IgM, IgA, IgG) responses over time were re-expressed as area under the curve (AUC) (MedCalc®) prior to performing the analyses. The mean qRT-PCR and antibody isotype AUCs for serum and oral fluid are given in Table 3. Statistical analysis (ANOVA) of main effects showed significant differences among trials (*p* < 0.001), sample type (*p* < 0.001), and quantitative responses (PRRSV, IgM, IgA, IgG; *p* < 0.001). With the exception of serum IgG, statistically significant differences in means were detected among trials both in the replication of PRRSVs and in antibody responses (Tukey’s Honestly Significant Difference (HSD) test). However, at the individual boar level, correlation analysis found a weak association between PRRSV viremia AUC and serum IgM, IgA, or IgG AUCs (r = 0.3762, 0.2915, and 0.0005). Likewise, the correlation was weak between PRRSV oral fluid AUC and oral fluid IgM, IgA, or IgG AUCs (r = 0.3147, 0.2671, and 0.2137).

**Table 3:** **Comparison of cumulative quantitative reverse transcription polymerase chain reaction (qRT-PCR) and antibody responses (IgM, IgA, IgG) for 21 days following PRRSV inoculation**^**1**^

	**Serum (mean AUC and 95% confidence intervals)**				**Oral fluid (mean AUC and 95% confidence intervals)**			
Trial (virus isolate)	qRT-PCR	IgM	IgA	IgG	qRT-PCR	IgM	IgA	IgG
Trial 1: (Ingelvac® PRRS MLV)	21.6^b^	46.6^b^	18.5^b^	17.4^a^	21.0^b^	27.6^b^	12.3^b^	25.1^b^
	(18.8-24.5)	(43.1-50.2)	(14.6-22.6)	(15.9-18.9)	(17.0-25.1)	(23.9-31.5)	(7.7-17.0)	(20.6-29.6)
Trial 2: (Type 1, D09-012332)	23.8^b^	39.5^b^	30.0^a^	18.3^a^	21.2^b^	17.6^c^	17.9^b^	21.9^b^
	(21.2-26.6)	(31.1-48.0)	(23.2-36.9)	(16.5-20.2)	(18.1-24.3)	(12.0-23.2)	(10.9-24.9)	(16.7-27.2)
Trial 3: (Type 2, MN-184)	38.1^a^	53.7^a^	31.6^a^	18.4^a^	31.6^a^	35.4^a^	26.2^a^	30.1^a^
	(34.9-41.5)	(48.1-59.3)	(24.7-38.5)	(17.1-19.85)	(28.4-34.9)	(28.6-42.3)	(19.1-33.5)	(25.3-34.9)

^1^ Individual boar PRRSV (log_e_ geq/μl) and antibody (IgM, IgA, IgG) S/P responses over time were summarized as the area under the curve (AUC) prior to performing the statistical analysis.

^abc^ Superscripts within columns indicate statistically significant differences among means (Tukey’s Honestly Significant Differences test, *p* < 0.05).

## Discussion

By definition, oral fluid is a mixture of saliva, oral mucosal transudate, and gingival crevicular fluid recovered from the buccal cavity using an absorptive device [12]. Among a variety of other constituents, oral fluid contains both locally-derived and systemic antibodies [13]. Thus, pathogen-specific IgM, IgA, and IgG for PRRSV [5], influenza A virus [7], and porcine circovirus type 2 [4] could be detected in oral fluid samples collected from groups of pigs (pens) under either experimental or field conditions. The largest proportion of locally produced antibody consists of dimeric secretory IgA (SIgA) produced by plasma cells in salivary glands and duct-associated lymphoid tissue (DALT) [14]. IgM and IgG are also produced in these tissues, but the majority of IgM and IgG in oral fluid is derived from serum via the gingival crevicular fluid [15].

In the present experiment, the collection of paired oral fluid and serum samples from 72 individually-housed boars inoculated with three different PRRSV isolates allowed for a more comprehensive evaluation of the onset and magnitude of serum and oral fluid antibody isotype responses, as well as the variation therein. Statistically significant differences in viral replication and antibody responses were observed among the three trials in both serum and oral fluid specimens (Table 3). Since each trial included only one virus isolate, statistically valid comparisons of the effects of virus isolates on PRRSV replication and antibody responses were not possible. Nevertheless, the statistically significant differences in virus replication and antibody response observed among trials was consistent with previous reports of virus isolate-dependent differences in the magnitude of replication in pigs [16] and corresponding differences in antibody response [16,17]. However, at the individual boar level, the correlation between virus replication and antibody response was relatively weak. Thus, what was true for the group of boars in a trial did not necessarily apply to an individual boar.

The purpose of this study was to describe and contrast the kinetics of PRRSV antibody in oral fluids and serum. PRRSV serum IgM, IgA, and IgG were first detected in samples collected on DPI 7, 10, and 10, respectively. These results were compatible with prior reports describing the detection of PRRSV serum IgM between DPI 5 and 7 [18-20], IgG between DPI 9 and 11 [19,21], and IgA at DPI 14 [19]. PRRSV oral fluid IgM, IgA, and IgG appeared concurrently with serum antibodies, but collection of daily oral fluid samples provided more precise estimates. That is, oral fluid IgM, IgA, and IgG were detected in samples collected between DPI 3 to 10, 7 to 10, and 8 to 14, respectively. There are no prior reports on PRRSV oral fluid antibody kinetics in individual animals with which to compare these results. However, we previously reported the detection of PRRSV oral fluid IgM and IgG in pen-based oral fluid samples from experimentally inoculated animals on DPI 7 [5]. Thus, the PRRSV serum and oral fluid antibody responses observed in this study were in agreement with prior observations on PRRSV and our general understanding of the humoral immune response as reflected in these sample matrices [14,22]. Most significantly, this study provided a broader foundation for understanding, developing, and interpreting oral fluid antibody-based diagnostics in the context of the humoral immune response.

## Conclusions

This study demonstrated that anti-PRRSV antibody isotypes can be detected in oral fluid specimens. These results were compatible with prior reports describing the detection of anti-PRRSV antibody in both serum and oral fluid. Detection of PRRSV antibody in oral fluids collected from individual boars could provide an effective approach for monitoring PRRSV infection in boar studs. Successful oral fluid collection and testing from individual boars suggests that approach could also be applied to population in swine production systems, i.e. pen-housed sows, farrowing crates, etc.

## Methods

### Experimental design

A total of 72 boars ranging from 6 months to 3.6 years of age under the ownership of PIC North America (Hendersonville, TN, USA) were used in this study. Housing, feed rations, animal care guidelines, and experimental protocols were approved and supervised by the PIC USA Health Assurance and Welfare department. In 3 trials of 24 boars each, animals were intramuscularly (i.m.) inoculated with either modified-live virus (MLV) PRRSV vaccine (Trial 1), a Type 1 field isolate (Trial 2), or a Type 2 field isolate (Trial 3). Serum and oral fluid samples were collected from all boars beginning 7 days prior to inoculation and continuing through 21 days post inoculation (DPI). After the completion of Trial 3, samples were completely randomized and tested for PRRSV IgM, IgA, and IgG antibody isotypes. Descriptive and comparative statistical analyses were conducted to describe and compare PRRSV antibody responses in serum and oral fluid and evaluate differences among individual boars and between trials.

### Animals and animal care

Boars were obtained from two Midwest USA breeding stock sources documented to be free of PRRSV infection. Culled boars (*n* = 24) ranged from one year to 3.6 years of age and select boars (*n* = 48) ranged from 5 to 6 months of age. The boars were housed in a commercial production facility equipped with nipple drinkers, concrete slatted flooring, curtains, and tunnel ventilation. Feeder space, water delivery, square footage per animal, sanitation, and ventilation parameters met or exceeded PIC North America health assurance and welfare requirements. Upon arrival, animals were housed individually in crates (Hog Slat, Inc., Newton Grove, NC USA) and fed a commercial corn/soy swine diet (Land O’ Lakes® Farmland Feed, Roland, IA USA) at a rate of 4 pounds per animal per day for acclimation/training and 7 pounds per animal per day thereafter.

### Porcine reproductive and respiratory syndrome viruses

In Trial 1, 24 boars were inoculated i.m. with 2 ml of a commercial MLV vaccine (Ingelvac® PRRS MLV, Boehringer Ingelheim Vetmedica, Inc., St. Joseph, MO 64506) rehydrated and administered according to the instructions provided by the manufacturer. In Trial 2, 24 boars were inoculated i.m. with 2 ml of a Type 1 PRRSV (isolate D09-012131) at an estimated concentration of 1 × 10^5.5^ median tissue culture infectious dose (TCID_50_) per ml. Isolate D09-012131 was isolated from serum samples submitted to the University of Minnesota Veterinary Diagnostic Laboratory (St. Paul, MN USA) in March 2009 as part of a routine monitoring program in a sow herd located in Illinois USA and propagated on pulmonary alveolar macrophage cells, as described elsewhere [11]. In Trial 3, 24 boars were IM inoculated with 2 ml of a Type 2 PRRSV isolate (MN-184, GenBank accession no. AY656992) at a concentration of 1 × 10^4.5^ TCID_50_ per ml. Isolate MN-184 (kindly provided by Dr. Scott Dee, University of Minnesota) was propagated on MARC-145 cells [23].

### Sample collection

#### Oral fluid collection

Oral fluid samples were collected daily from individually-housed boars beginning 7 days prior to inoculation and continuing through 21 days post inoculation (DPI) using a procedure described by Kittawornrat et al. [11]. In brief, oral fluid samples were collected by allowing boars to chew on 1.6 cm (5/8”) cotton rope (Web Rigging Supply, Inc., Lake Barrington, IL USA). Prior to collection, ropes were soaked with a solution of sucrose and apple juice (unsweetened apple juice with 50% (v/v) sucrose) and then air-dried. To collect oral fluid samples, ropes were placed in “rope holders” fixed at the front of each pen for 20 minutes. Oral fluids were deposited as the boars chewed on the rope. To recover the oral fluid specimens, the bottom 15 cm (~6”) of the rope (wet portion) was inserted into a 3.8 liter (one gallon) re-sealable plastic bag and severed from the dry portion of the rope. The bag with the wet rope inside was passed through a wringer (Dyna-Jet Products, Overland Park, KS USA), causing the fluid to pool in the bottom of the bag. Samples were then decanted into a 50 ml centrifuge tube and the volume recorded. Thereafter, samples were centrifuged at 1,000 × *g* for 10 minutes at 4°C, aliquoted into 5 ml plastic tubes (Becton, Dickinson and Company, Bedford, MA USA), and stored at −80°C until assayed.

#### Serum collection

In each trial, serum samples were collected from all boars on DPI −7, 0, 7, 14, and 21. Additional serum samples were collected on DPI 3, 5, 10, 17 from a subset of boars (*n* = 4) randomly selected at the beginning of each trial. Blood was collected by jugular venipuncture using serum separation tubes (Corvac®, Tyco Healthcare Group LP, Mansfield, MA USA). Samples were centrifuged at 1,000 × *g* for 10 minutes and the serum was aliquoted into 5 ml plastic tubes (Becton, Dickinson and Company) and stored at −80°C until assayed.

#### PRRSV antibody ELISAs

##### Commercial PRRSV serum antibody ELISA

All serum samples were assayed for PRRSV antibodies using a commercial indirect ELISA (PRRS X3 Ab Test, IDEXX Laboratories, Inc., Westbrook, ME USA) performed according to the manufacturer’s instruction. As recommended by the manufacturer, a positive result was defined as a sample-to-positive (S/P) ratio ≥ 0.4. Modifications to the commercial serum ELISA for the detection of antibody isotypes in serum and oral fluid are described below and listed in Table 4.

**Table 4:** **Summary of porcine reproductive and respiratory syndrome virus (PRRSV) serum and oral fluid antibody enzyme linked-immunosorbent assay (ELISA) condition**^**a**^

	**IgM**	**IgA**	**IgG**	**Commercial ELISA**
**Serum ELISAs**				
Sample dilution	1:40	1:5	1:40	1:40
Sample volume	100 μl	100 μl	100 μl	100 μl
Conjugate dilution	1:5,000^b^	1:1,000^c^	1:15,000^d^	Provided with kit
Negative control	100 μl of pooled negative serum diluted 1:40	100 μl of pooled negative serum diluted 1:5	100 μl of kit negative control	100 μl of kit negative control
Positive control	100 μl of pooled serum from DPI 7 diluted 1:40	100 μl of pooled serum from DPI 21 diluted 1:5	100 μl of kit positive control	100 μl of kit positive control
**Oral fluid ELISAs**				
Sample dilution	1:2	1:2	1:2	
Sample volume	250 μl	250 μl	250 μl	
Conjugate dilution	1:3,800^b^	1:2,000^c^	1:2,400^d^	
Negative control	250 μl of reference standard oral fluid^e^ DPI 0 diluted 1:2	250 μl of reference standard oral fluid^e^ DPI 0 diluted 1:2	100 μl of kit negative control diluted 1:30	
Positive control	250 μl of reference standard oral fluid^e^ DPI 10diluted 1:5	250 μl of reference standard oral fluid^e^ DPI 91 diluted 1:2	100 μl of kit positive control diluted 1:30	

^a^ Oral fluid ELISA conditions represent modifications to a commercial PRRSV serum antibody ELISA protocol (IDEXX PRRS X3 Ab Test, IDEXX Laboratories, Inc., Westbrook, ME, USA).

^b^ Anti-pig IgM: HRPO conjugate (A100-100P, Bethyl Laboratories, Montgomery, TX, USA).

^c^ Anti-pig IgA: HRPO conjugate (A100-102P, Bethyl Laboratories, Montgomery, TX, USA).

^d^ Anti-pig IgG_Fc_: HRPO conjugate (A100-104P, Bethyl Laboratories, Montgomery, TX, USA).

^e^ Reference standard oral fluid samples have been previously described [5].

##### PRRSV antibody isotypes in serum

The commercial indirect ELISA (PRRS X3 Ab Test) was modified to detect PRRSV-specific IgM, IgA, and IgG antibody isotypes in serum. In brief, serum samples were diluted 1:40 (5 μl serum sample + 195 μl kit diluent) for IgM and IgG and 1:5 (40 μl serum sample + 160 μl kit diluent) for IgA. 100 μl of diluted serum was then transferred to the PRRSV antigen-coated plates and incubated for 30 minutes at 22°C. After washing 3 times with 1X kit wash solution (400 μl), appropriately diluted horseradish peroxidase (HRPO)-conjugated anti-pig immunoglobulin (Ig) antibody (IgM (A100-100P), IgA (A100-102P), or IgG_Fc_ (A100-104P) (Bethyl Laboratories, Montgomery, TX USA) was added to each well and incubated for 30 minutes at 22°C. Thereafter, plates were washed three times with kit washing solution, after which 100 μl of tetramethylbenzidine (TMB) was added to each well and the plates incubated at 22°C for 15 minutes. At precisely 15 minutes, 100 μl of kit stop solution was added to each well. The plates were read at 650 nm using an ELISA plate reader (EL800 micro plate reader, Bio Tek® Instruments Inc., Winooski, VT) controlled by commercial software (Gen5™ Bio Tek® Instruments Inc., Winooski, VT USA) and the reactions measured as optical density (OD).

##### PRRSV antibody isotypes in oral fluid

Modification of the commercial PRRSV ELISA for the detection of PRRSV-specific IgM, IgA, and IgG antibody in swine oral fluid has previously been described [5]. In brief, oral fluid samples were diluted 1:2 (150 μl oral fluid sample + 150 μl kit diluent). 250 μl of diluted oral fluid was then transferred to PRRSV antigen-coated plates and incubated for 16 hours at 4°C. Thereafter, the plates were washed three times with 400 μl of 1X kit wash solution. To detect the reaction, 100 μl of a solution containing appropriately diluted HRPO-conjugated anti-pig Ig (M, A, or G) was added to each well and the plates incubated for 30 minutes at 22°C. The procedure for determining the optimal dilution of secondary antibody is described in preparation of secondary antibody section. After washing three times, 100 μl of TMB was added to each well and the plates incubated at 22°C for 15 minutes. Finally, 100 μl of kit stop solution was added to each well. As described in preparation of secondary antibody section, the plates were read at 650 nm and the reactions measured as optical density (OD).

#### Preparation of secondary antibody

**Figure 4:**
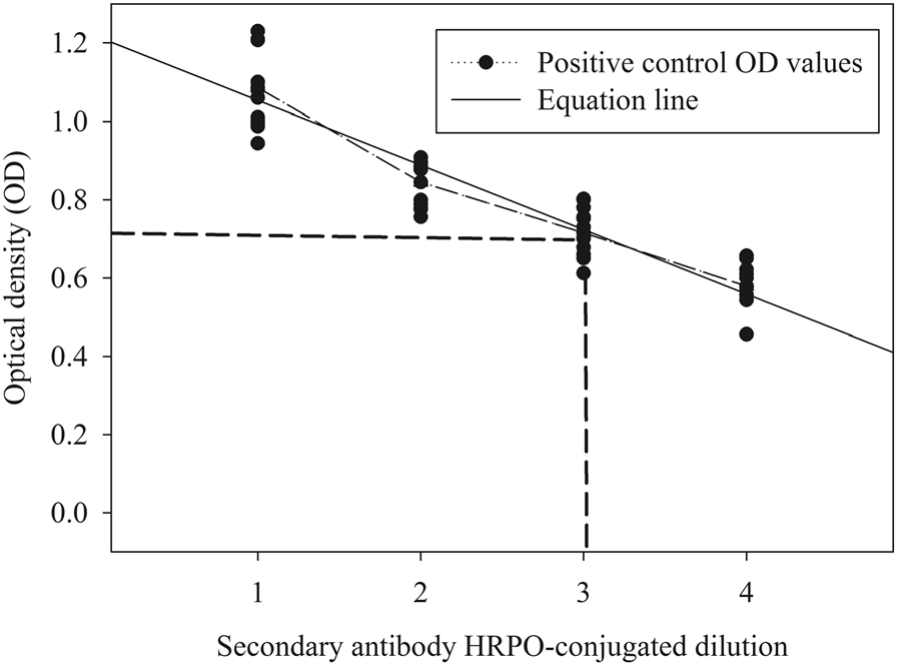
**Calculation of the optimal dilution of anti-pig secondary antibody for PRRSV IgM, IgA, IgG ELISAs.** The relationship between the positive control optical density (OD) and dilution of anti-pig secondary antibody was plotted as y = ax + c, where (y) is the secondary antibody OD response, (a) is the slope of the line, (x) is the dilution of secondary antibody, and (c) is the intercept. The appropriate dilution corresponds to the positive control value provided in the Certificate of Analysis.

To assure assay repeatability, the concentration of anti-pig Ig (M, A, or G) was standardized using the positive control OD value listed in the manufacturer’s Certificate of Analysis as the benchmark. The general procedure for calculating the conjugate dilution was as follows: 4 dilutions of anti-pig Ig (M, A, or G) were prepared in bottles wrapped in aluminum foil using diluent provided by manufacturer (IDEXX Laboratories, Inc., Westbrook, MA USA) and then stirred for 48 hours at 4°C. The reactivity of the 4 dilutions was determined using negative and positive kit controls. Specifically, kit negative control was dispensed into 48 wells (one-half plate) and kit positive control in each of the remaining 48 wells. Negative control OD values were used to screen for non-specific reactions and positive control OD values were used to determine the equation of the line:

(1) y=ax+c

where (y) is the anti-pig Ig (M, A, or G) OD response, (a) is the slope of the line, (x) is the dilution of anti-pig Ig (M, A, or G), and (c) is the intercept (Figure 4). Thereafter, the correct dilution of anti-pig Ig (M, A, or G) was calculated by substituting the mean positive control OD from the Certificate of Analysis for “y” in Equation 1 and solving for “x”.

The general procedure for preparing the appropriate dilution of anti-pig Ig (M, A, or G) was modified for serum and oral fluid antibody ELISAs. In this study, all serum and oral fluid specimens were tested on a single lot of ELISA kits. Thus, the appropriate dilution of anti-pig Ig was calculated specifically for the PRRSV indirect ELISA (PRRS X3 Ab Test) kit lot (#99-40959-W721).

For the serum ELISA, the correct dilution of anti-pig IgG_Fc_ was determined by titrating 4 dilutions of anti-pig IgG_Fc_ (1:14,000, 1:14,500, 1:15,000, 1:15,500) against 100 μl of kit positive control and generating the equation of the line, as described above. The appropriate dilution of anti-pig IgM was determined by titrating 4 dilutions of secondary antibody (1:4,000, 1:4,500, 1:5,000, 1:5,500) against 100 μl of a positive control consisting of a 1:40 dilution of a pool of serum from 72 boars at DPI 7. For anti-pig IgA, 4 dilutions (1: 1,000, 1:2,500, 1:3,000, 1:3,500) were titrated against 100 μl of a positive control consisting of a 1:5 dilution of a pool of serum from 72 boars at DPI 21. Diluted conjugate was simultaneously titrated against negative controls, i.e., kit negative control for anti-pig IgG_Fc_ and a pool of serum from 72 boars at DPI 0 for anti-pig IgA (1:5) and anti-pig IgM (1:40).

The protocol for preparing the optimal secondary antibody dilution for the PRRSV oral fluid ELISA has been described elsewhere [5]. To prepare anti-pig IgG_Fc_, 4 conjugate dilutions (1:1,000, 1:1,500, 1:2,000, 1:2,500) were titrated against 100 μl of kit negative and positive controls diluted 1:30 (10 μl kit control + 290 μl kit sample diluent). Controls for anti-pig IgM and anti-pig IgA consisted of oral fluid samples from PRRSV-negative pigs vaccinated with 2 ml of PRRS modified live virus (MLV) vaccine (Ingelvac® PRRS MLV, Boehringer Ingelheim Vetmedica, Inc., St. Joseph, MO USA). The sample collected immediately prior to vaccination was used as negative control. Samples collected on 10 and 56 days post vaccination were used as positive controls for anti-pig IgM and anti-pig IgA conjugates, respectively. Four dilutions of anti-pig IgM (1: 3,000, 1:3,500, 1:4,000, 1:4,500) and anti-pig IgA (1:1,000, 1:1,500, 1:2,000, 1:2,500) were used to calculate appropriate dilution, as described previously.

#### Quantitative reverse transcription polymerase chain reaction (qRT-PCR)

Detailed qRT-PCR protocols for serum and oral fluids are reported elsewhere [11,24]. In brief, nucleic acid extraction from serum and oral fluid samples was performed using a commercial RNA extraction kit (Ambion® MagMax™-96 Viral RNA isolation kit, Applied Biosystems™, Foster City, CA USA). Real-time PCR was performed with commercial reagent sets (TaqMAN® NA and EU PRRSV Reagents and TaqMAN® NA and EU PRRSV Controls, Applied Biosystems™) using the following cycling conditions: 1 cycle at 45°C for 10 minutes, 1 cycle at 95°C for 10 minutes, 40 cycles of: 97°C for 2 seconds, 60°C for 40 seconds. Eight 10-fold serially-diluted (10^0^ to 10^7^ copies/μl) plasmid-derived commercial standards (TaqMAN® NA and EU PRRSV RNA controls, Applied Biosystems™) were run on each PCR plate and their Ct values used to derive a standard curve. Samples were quantified as genome equivalents per μl (geq/μl) by fitting the sample Cts to the standard curve using the AB7500 Fast System SDS Software (Applied Biosystems™).

### Statistical analysis

All optical density (OD) data were converted to sample-to-positive (S/P) ratios prior to statistical analysis using the following formula:

(2) $$\text{S}/\text{P}=\left[\mathrm{Sample}\;\text{A}\left(650\right)–\mathrm{NC}\right]/\left(\mathrm{PC}–\mathrm{NC}\right)$$

where NC and PC represented the mean OD of the two negative control wells and two positive control wells, respectively. Statistical analyses were performed using SAS® Version 9.2 (SAS® Institute Inc., Cary, NC USA) and MedCalc® 12.3.0.0 (MedCalc Software, Mariakerke Belgium).

Initially, descriptive and comparative analyses were conducted to describe the onset, magnitude, and duration of PRRSV antibody isotype S/P ratios in serum and oral fluid. Thereafter, S/P results were analyzed in a linear mixed model with repeated measures (SAS® PROC GLIMMIX) using trial (1, 2, 3), DPI, boar age (month), oral fluid volume (ml), and their pairwise interactions as fixed effects and “boar” as the subject of repeated measures. Tukey’s Honestly Significantly Difference (HSD) test was used to detect statistically significant differences between S/P ratios in each trial by DPI. Pearson’s correlation coefficient (SAS® PROC CORR) was used to evaluate the overall quantitative relationship between IgM, IgA, and IgG S/P ratios in serum and oral fluid samples. Defining an S/P ratio ≥ 0.4 as positive [5], McNemar’s test (SAS® PROC FREQ) for paired samples was used to determine whether the proportion IgG ELISA positive serum and oral fluid samples were significantly different by trial (1, 2, 3) and DPI.

The association between the level of PRRSV replication and the strength of the humoral response was evaluated in individual boars. The virus concentration (log_e_ geq/μl) and antibody isotype S/P responses (IgM, IgA, IgG) in serum and oral fluid over the course of the experiment were re-expressed as the area under the curve (AUC; MedCalc®) and evaluated using

Pearson’s Correlation Coefficient (SAS® PROC CORR). In addition, the AUC data were evaluated for statistically significant differences among sample types (serum, oral fluid), trials (1, 2, 3), and quantitative responses (PRRSV, IgM, IgA, IgG) by analysis of variance (ANOVA). Sample type, trial, response, and their interaction were used as fixed effects in the model. Thereafter, Tukey’s Honestly Significant Differences (HSD) test was used to test for statistically significant differences among trial means.

## Competing interests

Authors A. Rice and S. Lizano are employed by IDEXX Laboratories, Inc. The remaining author(s) declare no conflicting interests with respect to their authorship or the publication of this article.

## Authors’ contributions

AK: data collection, antibody isotypes, manuscript preparation, and writing. ME: study conception, research design. YP: antibody isotypes, data collection. CO: antibody isotypes, data collection. KS: study conception, research design, and virus inoculation. AR: study conception. SL: study conception. CW: data analysis, study design. JZ: study design, manuscript preparation and writing. All authors read and approved the final manuscript.

## References

[B1] Madar R Straka S Baska T Detection of antibodies in saliva–an effective auxiliary method in surveillance of infectious diseases Bratisl Lek Listy 2002 103 38 41 12061087

[B2] Corthier G Swine fever: influence of passive immunity on pig immune response following vaccination with a live virus vaccine (Thiverval strain) Ann Rech Vet 1976 7 361 372 1028380

[B3] Corthier G Aynaud J Comparison of the immune response in serum and bucco-pharyngeal secretions following immunization by different routes with a live hog cholera virus vaccine (Thiverval strain) Ann Vet Res 1977 8 159 165 596794

[B4] Prickett JR Johnson J Murtaugh MP Puvanendiran S Wang C Zimmerman JJ Opriessnig T Prolonged Detection of PCV2 and Anti-PCV2 Antibody in Oral Fluids Following Experimental Inoculation Transbound Emerg Dis 2011 58 121 127 10.1111/j.1865-1682.2010.01189.x 21223532

[B5] Kittawornrat A Prickett J Wang C Panyasing Y Ballagi A Rice A Main R Johnson J Rademacher C Hoogland M Rowland R Zimmerman J Detection of porcine reproductive and respiratory syndrome virus (PRRSV) antibodies in oral fluid specimens using a commercial PRRSV serum antibody ELISA J Vet Diagn Invest 2012 24 262 269 10.1177/1040638711435679 22379043

[B6] Prickett J Simer R Christopher-Hennings J Yoon KJ Evans RB Zimmerman JJ Detection of Porcine reproductive and respiratory syndrome virus infection in porcine oral fluid samples: a longitudinal study under experimental conditions J Vet Diagn Invest 2008 20 156 163 10.1177/104063870802000203 18319427

[B7] Panyasing Y Goodell CK Wang C Kittawornrat A Prickett JR Schwartz KJ Ballagi A Lizano S Zimmerman JJ Detection of Influenza A Virus Nucleoprotein Antibodies in Oral Fluid Specimens From Pigs Infected Under Experimental Conditions Using a Blocking ELISA Transbound Emerg Disin press 10.1111/tbed.12019 23046061

[B8] De Buysscher E Berman D Secretory immune response in intestinal mucosa and salivary gland after experimental infection of pigs with transmissible gastroenteritis virus Am J Vet Res 1980 41 1214 1220 7192522

[B9] Loftager M Eriksen L Nielsen R Antibodies against Actinobacillus pleuropneumoniae serotype 2 in mucosal secretions and sera of infected pigs as demonstrated by an enzyme-linked immunosorbent assay Res Vet Sci 1993 54 57 62 10.1016/0034-5288(93)90011-4 8434149

[B10] De Buysscher E Dubois R Detection of IgA anti-Escherichia coli plasma cells in the intestine and salivary glands of pigs orally and locally infected with E. coli Adv Exp Med Biol 1978 107 593 600 10.1007/978-1-4684-3369-2_67 369316

[B11] Kittawornrat A Prickett J Chittick W Wang C Engle M Johnson J Patnayak D Schwartz T Whitney D Olsen C Schwartz K Zimmerman J Porcine reproductive and respiratory syndrome virus (PRRSV) in serum and oral fluid samples from individual boars: Will oral fluid replace serum for PRRSV surveillance? Virus Res 2010 154 170 176 10.1016/j.virusres.2010.07.025 20670665

[B12] Atkinson JC Dawes C Ericson T Fox PC Gandara BK Malamud D Mandel ID Navazesh M Tabak LA Guidelines for saliva nomenclature and collection Ann N Y Acad Sci 1993 694 xi xii

[B13] Ebersole JL Humoral immune responses in gingival crevice fluid: local and systemic implications Periodontol 2003 31 135 166 10.1034/j.1600-0757.2003.03109.x 12657000

[B14] Brandtzaeg PER Do Salivary Antibodies Reliably Reflect Both Mucosal and Systemic Immunity? Ann N Y Acad Sci 2007 1098 288 311 10.1196/annals.1384.012 17435136

[B15] Challacombe SJ Russell MW Hawkes J Passage of intact IgG from plasma to the oral cavity via crevicular fluid Clin Exp Immunol 1978 34 417 422 105828 PMC1537551

[B16] Johnson W Roof M Vaughn E Christopher-Hennings J Johnson CR Murtaugh MP Pathogenic and humoral immune responses to porcine reproductive and respiratory syndrome virus (PRRSV) are related to viral load in acute infection Vet Immunol Immunopathol 2004 102 233 247 10.1016/j.vetimm.2004.09.010 15507308

[B17] Martínez-Lobo FJ Díez-Fuertes F Segalés J García-Artiga C Simarro I Castro JM Prieto C Comparative pathogenicity of type 1 and type 2 isolates of porcine reproductive and respiratory syndrome virus (PRRSV) in a young pig infection model Vet Microbiol 2011 154 58 68 10.1016/j.vetmic.2011.06.025 21831539

[B18] Joo HS Park BK Dee SA Pijoan C Indirect fluorescent IgM antibody response of pigs infected with porcine reproductive and respiratory syndrome virus Vet Microbiol 1997 55 303 307 10.1016/S0378-1135(96)01332-6 9220626

[B19] Loemba HD Mounir S Mardassi H Archambault D Dea S Kinetics of humoral immune response to the major structural proteins of the porcine reproductive and respiratory syndrome virus Arch Virol 1996 141 751 761 10.1007/BF01718333 8645111 PMC7086943

[B20] Park BK Joo HS Dee SA Pijoan C E**valuation of an indirect fluorescent IgM antibody test for the detection of pigs with recent infection of porcine reproductive and respiratory syndrome virus** J Vet Diagn Invest 1995 7 544 546 10.1177/104063879500700422 8580181

[B21] Yoon KJ Zimmerman JJ Swenson SL McGinley MJ Eernisse KA Brevik A Rhinehart LL Frey ML Hill HT Platt KB Characterization of the humoral immune response to porcine reproductive and respiratory syndrome (PRRS) virus infection J Vet Diagn Invest 1995 7 305 312 10.1177/104063879500700302 7578443

[B22] Prickett JR Zimmerman JJ The development of oral fluid-based diagnostics and applications in veterinary medicine Anim Health Res Rev 2010 11 207 216 10.1017/S1466252310000010 20202287

[B23] Kim HS Kwang J Yoon IJ Joo HS Frey ML Enhanced replication of porcine reproductive and respiratory syndrome (PRRS) virus in a homogenous subpopulation of MA-104 cells Arch Virol 1993 133 477 483 10.1007/BF01313785 8257302

[B24] Chittick WA Stensland WR Prickett JR Strait EL Harmon K Yoon KJ Wang C Zimmerman JJ Comparison of RNA extraction and real-time reverse transcription polymerase chain reaction methods for the detection of porcine reproductive and respiratory syndrome virus in porcine oral fluid specimens J Vet Diagn Invest 2011 23 248 253 10.1177/104063871102300208 21398443

